# Regulation of Mitochondrial Quality Control by Natural Drugs in the Treatment of Cardiovascular Diseases: Potential and Advantages

**DOI:** 10.3389/fcell.2020.616139

**Published:** 2020-12-23

**Authors:** Xing Chang, Wenjin Zhang, Zhenyu Zhao, Chunxia Ma, Tian Zhang, Qingyan Meng, Peizheng Yan, Lei Zhang, Yuping Zhao

**Affiliations:** ^1^China Academy of Chinese Medical Sciences, Beijing, China; ^2^Guang’anmen Hospital of Chinese Academy of Traditional Chinese Medicine, Beijing, China; ^3^College of Pharmacy, Ningxia Medical University, Yinchuan, China; ^4^Shandong Analysis and Test Center, Qilu University of Technology, Jinan, China; ^5^Shandong University of Traditional Chinese Medicine, Jinan, China; ^6^Jiangxi University of Traditional Chinese Medicine, Nanchang, China

**Keywords:** natural drugs, effective active ingredients, mitochondrial quality control, cardiovascular diseases, oxidative stress, reactive oxygen species

## Abstract

Mitochondria are double-membraned cellular organelles that provide the required energy and metabolic intermediates to cardiomyocytes. Mitochondrial respiratory chain defects, structure abnormalities, and DNA mutations can affect the normal function of cardiomyocytes, causing an imbalance in intracellular calcium ion homeostasis, production of reactive oxygen species, and apoptosis. Mitochondrial quality control (MQC) is an important process that maintains mitochondrial homeostasis in cardiomyocytes and involves multi-level regulatory mechanisms, such as mitophagy, mitochondrial fission and fusion, mitochondrial energy metabolism, mitochondrial antioxidant system, and mitochondrial respiratory chain. Furthermore, MQC plays a role in the pathological mechanisms of various cardiovascular diseases (CVDs). In recent years, the regulatory effects of natural plants, drugs, and active ingredients on MQC in the context of CVDs have received significant attention. Effective active ingredients in natural drugs can influence the production of energy-supplying substances in the mitochondria, interfere with the expression of genes associated with mitochondrial energy requirements, and regulate various mechanisms of MQC modulation. Thus, these ingredients have therapeutic effects against CVDs. This review provides useful information about novel treatment options for CVDs and development of novel drugs targeting MQC.

## Introduction

Cardiovascular diseases (CVDs) are among the primary causes of death worldwide. With aging and lifestyle choices, CVDs have negatively affected humans’ health (Slovinski et al., 2019; Soler-Botija et al., 2019). In recent years, studies have shown that mitochondrial quality control (MQC) plays a key role in the treatment of CVDs (Li et al., 2020). Mitochondria are important semi-autonomous, double-membraned organelles, playing various regulatory roles in cell energy metabolism, signal transduction, reactive oxygen species (ROS) production, and apoptosis (Brooks, 2018; Kim D. H. et al., 2019). The mitochondria can determine the survival and death of cells, provide cellular energy through oxidative phosphorylation, and regulate the homeostasis of Ca^2+^, iron, and electrolytes (Tian L. et al., 2019). As the regulatory center of apoptosis, mitochondria can also release apoptotic factors following stimulation by apoptosis signals, trigger caspase-dependent or caspase-independent apoptosis pathways, and induce programmed cell death (Kordalewska and Markuszewski, 2015).

Mitochondria can meet the various physiological needs of myocardial cells upon changes in their physiological environment and form ATP through the tricarboxylic acidcycle and oxidative phosphorylation (Wang Y. et al., 2016; Yan S. et al., 2019), which provide energy for myocardial cell activity (Chen H. I. et al., 2020). In addition, the morphology and distribution of mitochondria are closely associated with the function of cardiomyocytes. Cardiomyocytes can be divided into two types: Inter-fiber mitochondria and sub-sarcosal mitochondria. The morphology of both types of mitochondria significantly changes with alterations in cardiomyocytes, including under pathological conditions. These morphological changes consist primarily of mitochondrial fission and fusion (Jong et al., 2019). If the stress state leads to a mitochondrial fission / fusion imbalance, mitochondrial function is then impaired, leading to inefficient aggregation, thereby impairing the metabolic capacity of cardiomyocytes (Pennanen et al., 2014).

As cardiomyocytes are highly dependent on mitochondria and cannot proliferate, they are not regulated by mitosis. Therefore, MQC plays an important role in maintaining the homeostasis of mitochondria and ultimately the normal physiological function of cardiomyocytes (Wang et al., 2020a). Mitochondria are very sensitive to nutrient and oxygen supply and can adapt to metabolic changes in different environments. In several CVDs cases, dysfunction of the respiratory electron transport chain in cardiomyocytes, abnormal ATP synthesis, or increased levels of mitochondrial oxidative stress dysregulate MQC, leading to the loss of mitochondrial structure integrity (Xue et al., 2017).

Furthermore, uncoupling of the electron transport chain in dysfunctional mitochondria increases the production of ROS, and depletion of ATP in cardiomyocytes accelerates their apoptosis (Song and Li, 2019). Previous studies have highlighted the importance of MQC in the future clinical treatments ofCVDs (Zhang Y. et al., 2019), with recent studies focusing on natural antioxidants. Natural antioxidants can be isolated and extracted from natural plants and can be used to effectively regulate MQC (Huang J. et al., 2018; Zhou Z. et al., 2018; Li Y. et al., 2019), particularly in the treatment of CVDs (Popova et al., 2018; Shakeri et al., 2020). In this review, we first discuss the regulation of MQC by active components in natural drugs, and then focused on the mechanism of various natural drugs and effective active ingredients for treating CVDs based on MQC regulation. This review provides an overview of natural drugs with potential for treating CVDs and to support further research in this field.

## MQC Regulatory Mechanisms in Cardiomyocytes

Mitochondrial quality control is an important process that maintains mitochondrial homeostasis in cells and primarily regulates mitochondrial quantity and quality. MQC includes the following mechanisms: Mitochondrion fission and fusion, a key mechanism in the dynamic control and repair of mitochondrial quality; mitophagy, which together with mitochondrial biosynthesis promote the degradation and renewal of mitochondria, respectively; mitochondrial antioxidant enzyme system, an important defense line against mitochondrial damage that maintains the “oxidation and anti-oxidation” balance in the cell; mitochondrial energy metabolism system, which provides energy for mitochondria and cardiac cell organelles via oxidative phosphorylation; mitochondrial respiratory chain, the “production line” of mitochondrial energy supply (Ni et al., 2015). Through these different mechanisms, which will be further discussed in the following subsections, MQC can ensure the regulation of both the quantity and quality of mitochondria in cardiomyocytes to safeguard mitochondrial defense, repair, and removal (Anzell et al., 2018; Thai et al., 2019; Wang et al., 2020a).

### Mitochondrial Fission and Fusion

Mitochondrial fission and fusion are the basis of MQC and are primarily mediated by GTPase. The mechanism of mitochondrial fusion is complex and divided into two parts: Mitochondrial outer membrane fusion and intimal fusion (Catanzaro et al., 2019a). Mitochondrial fusion promotes internal material exchange in mitochondria, accelerates the repair of damaged mitochondrial genes, and maintains mitochondrial integrity. Under stress conditions, slightly damaged mitochondria can fuse with the mitochondrial network, resist the interference of stress factors due to the stability of the mitochondrial network, and maintain ATP synthesis (Sharp and Archer, 2015).

Unlike fusion, mitochondrion fission involves only regulators of mitochondrial outer membrane division. During which the mitochondrion can produce two mitochondria with unbalanced membrane potentials: That with a normal membrane potential fuses with the mitochondrial network and participates in the cycle of mitochondrial network fission and fusion, whereas that with a lower membrane potential can be selectively removed by mitophagy to ensure homeostasis of the internal mitochondrial environment. Therefore, mitochondrion fission also acts as the first step in the process of mitophagy (Murata et al., 2020).

Mitochondrial fission and fusion are frequent and extensive mechanisms of mitochondrial self-protection in cells, and the dynamic balance between these processes determines the number, morphology, and distribution of mitochondria to meet the different physiological needs of cardiomyocytes.

### Mitophagy

Mitophagy is the primary mechanism regulating mitochondrial energy metabolism, self-repair, and renewal (Lazarou et al., 2015; Campos et al., 2017). Mitophagy is a selective autophagy that targets mitochondria and represents the main pathway of autophagy in cardiomyocytes (Li Y. Z. et al., 2019; Tian Y. et al., 2019). The autophagy precursor with a double-layer membrane can specifically wrap senescent and damaged mitochondria present in the cell and fuse with lysosomes to form autophagic lysosomes (Hughes et al., 2020). Through the recycling and reuse of mitochondrial components, self-renewal is achieved, maintaining mitochondrial homeostasis (Li et al., 2018). Therefore, weakened autophagic activity can exacerbate mitochondrial oxidative damage, leading to irreversible damage, the accumulation of mitochondria, and acceleration of cardiomyocyte apoptosis (Li J. et al., 2019; Xiong et al., 2019).

Under oxidative stress, cardiomyocytes can also reduce ROS production by activating mitophagy, prevent the opening of mitochondrial permeability transition pores (mPTPs), improve mitochondrial quality, and ensure their basic energy requirements (Tian L. et al., 2019; Villarejo-Zori et al., 2020). If mitophagic activity is reduced, damaged mitochondria clear obstacles in the cell and accumulate excessively, causing severe oxidative stress damage. In contrast, if mitophagy is excessive, the number of mitochondria in cardiomyocytes decreases, mitochondrial energy metabolism is impaired, and cardiomyocyte apoptosis is accelerated (Zha et al., 2017; Catanzaro et al., 2019a).

### Mitochondrial Energy Metabolism

Mitochondrial energy metabolism dysfunction caused by mitophagy dysfunction and impaired electron transport chain can induce cardiomyocyte apoptosis, leading to irreversible cell damage due to acute ischemia and hypoxia (Sun and Yang, 2017a). For example, myocardial cell apoptosis caused by myocardial ischemia-reperfusion (I/R) injury is related to mitochondrial energy metabolism disorder (Boenzi and Diodato, 2018; Tian L. et al., 2019). This occurs since stress-induced mitochondrial energy metabolism disorder promotes the aggregation of B-cell lymphoma 2 (Bcl-2) family proteins (Bcl-2 associated X, apoptosis regulator (Bax)/Bcl-2-like protein 4 (Bak)) on mitochondria, in turn leading to the formation of pores in their outer membrane. Through these open channels, apoptotic factors in mitochondria, such as cytochrome C, bind to apoptotic protease activating factor-1 and activate caspase-9. The cascade of pro-apoptotic proteases leads to cardiomyocyte apoptosis (Liu X. et al., 2017).

In addition, abnormal opening of mPTPs caused by mitochondrial energy metabolism disorder can lead to the release of cytochrome C and promote ion exchange between the mitochondrial matrix and cytoplasm, resulting in mitochondria swelling and deformation, as well as oxidative phosphorylation collapse and cell necrosis (Dorn, 2010). Furthermore, the mitochondrial energy metabolism function also influences the regulation of mitochondrial respiratory chain, which together maintain the energy supply of myocardial cells.

### Mitochondrial Respiratory Chain

The mitochondrial respiratory chain is the primary pathway for ATP synthesis (Anso et al., 2017). Under normal physiological conditions, more than 98 % of transferred electrons are effectively used to synthesize ATP in the mitochondrial respiratory chain. The remaining electrons are released into the cytoplasm to produce low levels of ROS, and superoxide radicals are transformed by superoxide dismutase (SOD) to prevent oxidative stress damage to mitochondria due to excessive accumulation of ROS. Once mitochondrial respiratory chain dysfunction occurs, uncoupling the electron transport chain from ATP leads to excessive production of ROS, which destroys mitochondrial DNA, lipids, and proteins (Duberley et al., 2013; Sommer et al., 2016).

Also, during myocardial I/R, mitochondrial respiratory chain function is impaired, leading to a rapid increase in ROS production and continuous opening of mPTPs, and induces activation of the cardiomyocyte apoptosis pathway (Ahmad et al., 2019b; Li J. et al., 2019). Therefore, activating endogenous mechanism associated with MQC to improve mitochondrial respiratory chain function and repair mitochondrial damage is an important strategy for protecting myocardial cells.

### Mitochondrial Antioxidant System

The mitochondrial respiratory chain is not only the primary site of ATP synthesis but also of ROS production (Gabrielova et al., 2010). Accumulation of ROS causes lipid peroxidation, abnormaloxidative phosphorylation, and mitochondrial damage (Huang J. et al., 2018; Kim Y. R. et al., 2019). In turn, the mitochondrial antioxidant system responds to the redox signal produced by ROS as a messenger molecule, participates in the regulation of cell signal transduction (Kowaltowski, 2019), and sustains the “oxidation and antioxidation” balance, which is extremely important for maintaining the quality of mitochondria (Wang L. et al., 2018; Zhu et al., 2019).

The antioxidant system of mitochondria is primarily composed of SOD, glutathione peroxidase, catalase, peroxidase reductase, and coenzyme (Oyewole and Birch-Machin, 2015; Ding et al., 2019). During myocardial I/R, the myocardial cell damage observed is attributed to the mitochondrial antioxidant system imbalance. When myocardial ischemia occurs, H^+^ leakage from the electron transport chain complex thereby damaging the electron transport chain and changing the mitochondrial membrane potential (MMP). The transient increase in oxygen concentration leads to the abrupt formation of ROS, making it much more challenging for the mitochondrial antioxidant system to adjust the “oxidation and anti-oxidation” balance which ultimately causes significant damage to the mitochondrial membrane (Du and Ko, 2006; Chen et al., 2008). Therefore, the mitochondrial antioxidant system represents the “antioxidant defense line” of MQC. Notably, drug research on CVDs treatment has always focused on regulation of the mitochondrial antioxidant system.

## Regulation of MQC by Natural Drugs

As shown in Table 1, active components of natural drugs can affect the production of energy-supplying components in mitochondria by interfering with the expression of genes associated with mitochondrial energy demand, as well as effectively regulating mitochondrial fission and fusion, mitochondrial energy metabolism, the mitochondrial antioxidant system, mitophagy, mitochondrial calcium homeostasis, and mPTPs (Liu X. et al., 2017; Chen et al., 2019; Oh et al., 2019). This illustrates the great potential of active components of natural drugs in the clinical treatment of CVDs. In the next subsections, we describe their mechanisms in regulating MQC.

**TABLE 1:** Effects of MQC on cardiomyocytes and regulatory mechanisms by natural drugs.

No	MQC	Physiological regulation of mitochondria and myocardial cells	Natural drugs	Regulation mechanism of MQC
1	Mitochondrion fission/fusion	Meets the physiological needs of cardiomyocytes in different environments	(1) Que (2) Baicalin (3) Res (4) ICA (5) Ginsenoside Rg5	(1) Inhibition of ROS in mitochondria (2) Inhibition of adenylate-dependent protein kinase (3) Inhibition of dynamic related protein 1 expression
2	Mitochondrial autophagy	Maintains the quantity and quality of myocardial mitochondria	(1) CTL (2) Salidroside (3) PNS (4) AST	(1) Improvement of mitochondrial biosynthesis (2) Activation of hypoxia-inducible factor-1α/Bcl-2 protein-interacting protein 3 (3) Inhibition of mitochondrial motility-related protein-1 and mitochondrion-1 expression
3	Mitochondrial energy metabolism	Maintains the energy supply of cardiomyocytes; it is the central link of MQC regulation	(1) AST (2) Ginsenoside	(1) Promotion of mitochondrial tricarboxylic acid cycle (2) Inhibition of mitochondrial unidirectional Ca^2+^ transporter activity (3) Stimulation of cell metabolic enzymes to synthesize ATP
4	Mitochondrial respiratory chain	Main pathway of ATP synthesis; leads to oxidative stress damage of mitochondria and cardiomyocytes	(1) Rubia cordifolia (2) Anthocyanins	(1) Increase in the activity of mitochondrial respiratory chain enzymes (2) Improvement of electron leakage (3) Inhibition of MDA activity
5	Mitochondrial antioxidant system	Defense mechanism that prevents oxidative stress injury of mitochondria and cardiomyocytes	(1) Que (2) Tea polyphenols (3) *Morinda* (4) Triterpenoid glycosides from black cohosh (5) Anthocyanin	(1) Increase in the expression of antioxidant enzymes (2) Inhibition of ROS production in mitochondria (3) Increase in the activity of SOD and glutathione peroxidase
6	mPTP	Regulates the calcium content of mitochondrial matrix; maintains homeostasis of the mitochondrial environment	(1) Ginsenoside Rg5 (2) Que (3) Capsaicin	(1) Inhibition of the abnormal opening of mPTP (2) Upregulation of silent information regulator 1 and Bcl-2 expression
7	Mitochondrial calcium homeostasis	Regulates the coupling ability between mitochondrial dehydrogenase and electron transfer complex; regulates the energy generation of mitochondria	(1) Dihydroartemisinin (2) Stevioside (3) Geniposide	(1) Regulation of mitochondrial Ca^2+^ unidirectional transporter protein (2) Promotion of endoplasmic reticulum Ca^2+^-ATPase activity

*ROS, reactive oxygen species; ATP, Adenosine triphosphate; MDA, Malondialdehyde; SOD, superoxide dismutase; mPTPs, mitochondrial permeability transition pores; Bcl-2, B-cell lymphoma 2.*

### Regulation of Mitochondrial Fission and Fusion

Mitochondria are highly dynamic organelles that can form branched or tubular network structures through continuous fusion and fission. The mechanisms of fusion and fission can also affect the shape and number of mitochondria. Quercetin (Que), resveratrol (Res), and icariin (ICA) inhibit excessive mitosis induced by ROS or cAMP-dependent protein kinase in mitochondria and maintain a normal quantity and quality of mitochondria (Liu et al., 2014; Li et al., 2016). In addition, ginsenoside Rg5 can reduce mitochondrion fission and improve isoproterenol-induced myocardial ischemia by inhibiting mitochondrial collection, mediated by dynamin-related protein 1 (Drp1), and participates in the interaction between mitochondria and the endoplasmic reticulum (Yang et al., 2017a).

### Regulation of Mitochondrial Energy Metabolism

The process of electron transfer in the mitochondrial electron transport chain is coupled with ATP production. Deficiency in mitochondrial bioenergy results in insufficient ATP production, which significantly affects the physiological function of cardiomyocytes (Cogliati et al., 2018; Guo et al., 2018). Astragaloside IV (AST) and active components of ginseng and *Ophiopogon japonicus* can promote the tricarboxylic acid cycle and improve the synthesis of mitochondrial bioenergy (Xu and Dou, 2016). AST can inhibit unidirectional Ca^2+^ transporter activity in mitochondria, reduce the level of mitochondrial Ca^2+^, stimulate cell metabolic enzymes to synthesize ATP, interfere with genes and signal pathways associated with mitochondrial energy demand, and regulate mitochondrial energy metabolism function (Xu and Dou, 2016; Dong et al., 2017). Furthermore, anthocyanins can be used as substrates of mitochondrial respiratory chain enzyme I, which can prevent respiratory chain enzymes from being inhibited by ischemia, maintain mitochondrial respiratory function, and protect myocardial cells from ischemia injury (Skemiene et al., 2015).

### Regulation of the Mitochondrial Antioxidant System

Natural drugs can also regulate the mitochondrial antioxidant system and reduce the effect of oxidative stress on mitochondrial quality. Que, tea polyphenols, and *Morinda* can increase the expression of antioxidant enzymes and inhibit mitochondrial ROS through nuclear factor erythroid 2-related factor 2 (Nrf2)-nucleolin recognition element signaling, as well as eliminate excessive ROS in cells and improve cell damage (Ballmann et al., 2015). Astragaloside-IV, triterpenoids, and anthocyanin in mulberry fruit can increase the activity of SOD and glutathione peroxidase and inhibit the production of ROS in mitochondria (Lee et al., 2016; Yang et al., 2016). Capsaicin can significantly reduce ROS production, inhibit the opening of mPTPs and caspase-3 activation, downregulate the expression of Bax, upregulate the expression of 14-3-3 eta and Bcl-2, reduce lactate dehydrogenase (LDH) release in H9c2 cells under hypoxia reoxygenation, and improve the viability of cardiomyocytes (Huang B. et al., 2018). Overall, by regulating ROS production, natural drugs can inhibit cell damage caused by oxidative stress.

### Regulation of Non-specific Mitochondrial Permeability Transition Pores (mPTPs)

The mPTPs are a non-specific channel regulated by the mitochondrial matrix calcium content and ROS through cyclophilin (Wang et al., 2015) and is closely associated with mitochondrial dysfunction. Abnormal mPTPs openings can cause apoptosis (Ma and Liu, 2019; Ahmad et al., 2019b). Ginsenoside Rg5 can inhibit mPTPs opening, reduce the sensitivity of mPTPs to external stimuli, and increase the resistance of myocardial cells to hypoxia/reoxygenation (H/R) injury. Moreover, Que was found to inhibit Ca^2+^-triggered mPTPs opening in the heart of rats with aldosteronism and interact with molecular targets in mitochondria, thus inhibiting the opening of these channels (De Marchi et al., 2009). Capsaicin was shown to improve H/R-induced mitochondrial dysfunction by upregulating the expression of silent information regulator 1 (SIRT1) and Bcl-2 and by inhibiting mPTPs opening (He et al., 2017).

Interestingly, other studies have shown that the transient opening of mPTPs may be a protective mechanism against mitochondrial calcium overload. The transient opening of mPTPs has been observed in primary myocardial mitochondria (Lu et al., 2016). Whether active ingredients in natural drugs can regulate the opening and closing of mPTPs warrant further investigations.

### Regulation of Mitophagy

Natural drugs and active ingredients can also regulate MQC by modulating mitophagy, which may become a new strategy in the treatment of CVDs (Feng et al., 2017; Lo et al., 2020a). Catalpol (CTL) increases myocardial mitophagy induced by glucose starvation and plays a protective role in the cardiomyocytes. Salidroside can enhance mitochondrial activity, activate autophagy, and mitochondrial biosynthesis in the myocardium, improve mitophagy levels, and improve stress injury of the skeletal muscle and myocardium in mice (Dun et al., 2017). In addition, *Panax notoginseng* saponins (PNS) were found to induce mitophagy by activating the hypoxia-inducible factor-1α/Bcl-2/beclin-1 signaling pathway and to reduce the toxicity of cisplatin (Liang W. Z. et al., 2017). AST can also significantly reduce the expression of mitochondrial motility-related protein-1, thus inhibiting mitophagy-induced by kinase 1/parkin (Liu X. et al., 2017).

### Regulation of Mitochondrial Calcium Homeostasis

As a local intracellular Ca^2+^ buffer, the mitochondrion can rapidly absorb a large amount of calcium ions, prevent excessive increases in intracellular Ca^2+^ levels, and inhibit calcium overload (Patron et al., 2018). The level of Ca^2+^ in the mitochondria determines the coupling ability between mitochondrial dehydrogenase and the electron transfer complex, regulating energy generation in mitochondria. Natural drugs can directly or indirectly regulate the interaction between mitochondrial Ca^2+^ transporters and endoplasmic reticulum Ca^2+^-ATPase and interfere with intracellular calcium homeostasis (Giorgi et al., 2012). Dihydroartemisinin was reported to regulate unidirectional Ca^2+^ transporter activity in mitochondria (Luo et al., 2018). In addition, stevioside and geniposide were found to promote endoplasmic reticulum Ca^2+^-ATPase activity, inhibit mitochondrial calcium overload, and regulate mitochondrial calcium homeostasis (Gao et al., 2017).

In summary, active components of natural drugs can regulate MQC through several mechanisms, providing new targeted therapeutic strategies for CVDs.

## Regulation of MQC by Natural Drugs in Different CVDs

**FIGURE 1:**
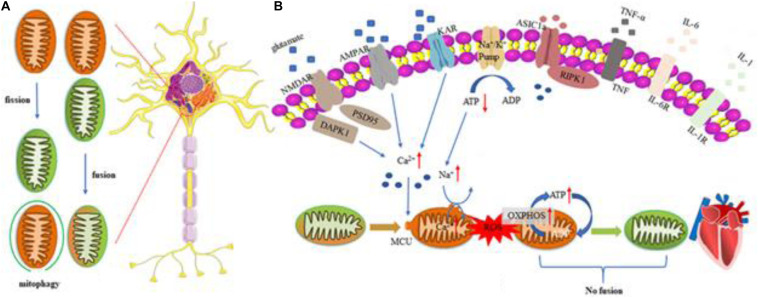
Regulation mechanism of MQC in cardiovascular disease. Mitochondrial response includes excessive ROS production, mitochondrial calcium overloading, and disrupted MQC. Excessive glutamate release and impeded reuptake of excitatory amino acids result in the activation of NMDARs, AMPARs, and KARs, as well as inflammatory reaction. Microglia are activated and release cytokines and chemokines to induce inflammation reaction. All factors mentioned above function synergistically to trigger cell death pathways such as apoptosis, necroptosis, and autophagy. **(A)** Regulatory mechanisms of Mitochondrial fusion/fission and mitophagy in cells. **(B)** Heart impaired mitophagy resulting in the development of dilated cardiomyopathy. Myocardial cells can reduce ROS production by activating mitochondrial autophagy, prevent mitochondrial permeability transition pores (mPTPs) from opening, improve mitochondrial quality and ensure its basic energy requirements. If the level of mitochondrial autophagy is reduced, the damaged mitochondria will clear away the obstacles in the cells and excessively accumulate, which will induce myocardial apoptosis and damage. ROS, reactive oxygen species; AMPAR, α-amino-3-hydroxy-5-methyl-4-isoxazole-propionic acid receptor; NMDAR, N-methyl-D-aspartate receptor; KAR, kainite receptor; DAPK1, death associated protein kinase 1; PSD95, postsynaptic density protein 95; ASIC1a, acid-sensing ion channel 1a; RIPK1, receptor interacting protein kinase 1; TNF-α, tumor necrosis factor-α; IL-6, Interleukin 6; IL-1, Interleukin 1; OXPHOS, oxidative phosphorylation.

As described above, natural drugs can control the quality of mitochondria by regulating their fission/fusion and energy metabolism, and autophagy, maintaining normal mitochondrial function (Takanashi et al., 2017; Arauna et al., 2019). As such, the effect of natural drugs on mitochondrial function has been investigated in previous studies on the material basis of natural drugs (Zhou H. et al., 2018; Lo et al., 2020a). As shown in Figure 1 and Table 2, various natural drugs and active ingredients can protect mitochondrial function and structure by regulating MQC, and their effectiveness has been preliminarily verified in experimental studies of coronary atherosclerotic heart disease (CHD), acute myocardial infarction (AMI), heart failure (HF), myocardial I/R injury, and other CVDs.

**TABLE 2:** Regulatory mechanism of MQC by different natural drugs in CVDs.

No	Bioactive ingredient	Original plant	Chemical name	Regulation mechanism of MQC	Targeting pathway	Target disease	Experimental objectives
1	Ginsenoside Rg5	*Panax*, etc.	C_41_H_68_O_12_	(1) Increase in HK-II binding to mitochondria (2) Inhibition of Drp1 recruitment (3) Activation of the Akt signaling pathway (4) Inhibition of mPTP opening	(1) Akt (2) Drp1 (3) HK-II	(1) Coronary heart disease	*In vitro*
2	Ginsenoside Rb1	*Panax*, etc.	C_54_H_92_O_23_	(1) Inhibition of cytochrome C transport (2) Increase in the level of mitochondrial transmembrane potential (3) Increase in the Bcl-2/Bax ratio (4) Inhibition of caspase-9 and caspase-3 activity	(1) Bcl-2/Bax	(1) Coronary heart disease	*In vitro*
3	BBR	*Coptis*, etc.	C_20_H_18_NO_4_	(1) Inhibition of cytochrome C transport (2) Decrease in MMP loss (3) Inhibition of AMPK-α and p53 phosphorylation (4) Inhibition of caspase-9 and caspase-3 activity	(1) Bcl-2/Bax (2) AMPK (3) p53	(1) Coronary heart disease	*In vitro* / *in vivo*
4	ORI	*Polygonum orientale*	C_21_H_20_O_11_	(1) Inhibition of abnormal mPTPs opening (2) Inhibition of cytochrome C transport (3) Increase in the Bcl-2/Bax ratio (4) Decrease in MMP loss (5) Inhibition of ROS production in mitochondria	(1) PI3K/Akt2 (2) Bcl-2/Bax	(1) Coronary heart disease	*In vitro*
5	PAE	*P. americana*	–	(1) Inhibition of cTNI and CK-MB overexpression (2) Inhibition of IL-1β, IL-6, and TNF-α overexpression (3) Promotion of mitochondrial autophagy (4) Increase in mitofusin 1, mitofusin 2, OPA1, and Drp1 expression	(1) PINK1/parkin (2) Mitofusin 1/Mitofusin 2 (3) Opa1/Drp1	(1) Coronary heart disease	*In vitro*
6	Que	*Crataegus pinnatiida*, *Hippophae rhamnoides*, etc.	C_15_H_10_O_7_	(1) Increase in the Bcl-2/Bax ratio (2) Inhibition of abnormal mPTP opening (3) Increase in Drp1 expression (4) Increase in ATP levels and MMP	(1) Drp1 (2) Bcl-2/Bcl-X	(1) AMI (2) Vascular calcification	*In vitro*
7	Tanshinone	*S. miltiorrhiza*	C_18_H_12_O_3_	(1) Reduction of MDA (2) Inhibition of ROS and LDH production (3) Enhancement of SOD activity in mitochondria (4) Decrease in NO and Ca^2+^ levels (5) Inhibition of abnormal mPTPs opening	(1) SIRT1-PGC1α	(1) AMI (2) I/R	*In vitro* / *in vivo*
8	Gas	*G. elata*	C_13_H_18_O_7_	(1) Inhibition of abnormal mPTPs opening (2) Inhibition of ROS production (3) Inhibition of mitochondrial respiratory function and expression of Mfn2 and OPA1 (4) Promotion of Nrf2 nuclear transport	(1) Mfn2 (2) Opa1 (3) Nrf2	(1) AMI	*In vitro*
9	AST	*Astragalus propinquus*	C_41_H_68_O_14_	(1) Inhibition of abnormal mPTPs opening (2) Inhibition of ROS/LDH/CK-MB production (3) Increase in MMP levels (4) Inhibition of cytochrome C transport	(1) Bcl-2 (2) PI3K/Akt (3) GSK-3β	(1) AMI (2) I/R	*In vitro* / *in vivo*
10	Ligustrazine	*Ligusticum chuanxiong*	C_8_H_12_N_2_	(1) Inhibition of abnormal mPTPs opening (2) Increase in MMP levels (3) Inhibition of LDH production (4) Inhibition of caspase-3 activity	(1) 14-3-3γ (2) Bcl-2 (3) Bad (S112)	(1) AMI	*In vitro*
11.	Res	*Veratrum grandiflorum*, etc.	C_14_H_12_O_3_	(1) Increase in LC3-II expression (2) Inhibition of caspase-3 activity (3) Increase in the Bcl-2/Bax ratio (4) Inhibition of ROS production	(1) Drp1 (2) parkin/PINK1 (3) SIRT1 (4) PGC-1α	(1) HF	*In vitro* / *in vivo*
12	PNS	*P. notoginseng*	–	(1) Increase in FOXO3a and Mn-SOD activity (2) Inhibition of MDA activity (3) Increase in LC3-II/Beclin-1 expression	(1) PGC-1α (2) Beclin-1	(1) HF	*In vitro* / *in vivo*
13	LTL	*Lonicera japonica*, etc.	C_15_H_10_O_6_	(1) Inhibition of ROS production (2) Increase in MMP level (3) Increase in LC3-II expression	(1) Drp1 (2) TFEB (3) LAMP1	(1) HF	*In vitro*
14	CTL	*Rehmannia glutinosa*	C_15_H_22_O_10_	(1) Increase in SOD activity (2) Inhibition of MDA activity (3) Inhibition of caspase-3 activity (4) Increase in the Bcl-2/Bax ratio	(1) Bcl-2/Bax	(1) HF	*In vitro*
15	DSG	*Dioscorea oppsite*	C_27_H_42_O_3_	(1) Blockage of the mitokatap channel and NO system (2) Inhibition of LDH release (3) Inhibition of IL-6/IL-1β/TNF-α expression	(1) mitokatap	(1) I/R (2) Arrhythmia	*In vitro* / *in vivo*
16	LYP	*Solanum lycopersicum, Punica granatum*, etc.	C_40_H_56_	(1) Inhibition of abnormal mPTP opening (2) Inhibition of cytochrome C transport (3) Inhibition of ROS and MDA activity (4) Increase in the Bcl-2/Bax ratio	(1) Bcl-2/Bax (2) APAF-1 (3) Tfam	(1) I/R (2) CHD	*In vitro*
17	Cur	*Curcuma longa*	C_21_H_20_O_6_	(1) Inhibition of ROS formation (2) Inhibition of MDA/hydrogen peroxide activity (3) Increase in MMP level (4) Increase in SOD activity in mitochondria	(1) SIRT1 (2) Bcl-2/Bax	(1) I/R (2) CHD	*In vitro* / *in vivo*
18	ICA	*Epimedium brevicomu, E. sagittatum*, *E. koreanum, E. pubescens*	C_33_H_40_O_15_	(1) Inhibition of MDA formation (2) Increase in SOD activity in mitochondria (3) Increase in MMP level (4) Inhibition of cytochrome C transport	(1) Sirtuin-1/Ac-FOXO1 (2) Bcl-2/Bax (3) p53	(1) I/R (2) CHD (3) hypertension	*In vitro* / *in vivo*

*Drp1, dynamin-related protein 1; HK-II, Hexokinase II; AMPK, Adenosine 5′-monophosphate (AMP)-activated protein kinase; PI3K, Phosphatidylinositol 3-kinase; PINK1, PTEN induced putative kinase 1; Mfn, Mitofusin; Opa1, Optic Atrophy 1; SIRT1, Sirtuin-1; PGC1α, peroxisome proliferator-activated receptor γ coactiva-tor-1; Nrf2, nuclear factor erythroid 2-related factor 2; GSK-3β, Glycogen synthase kinase 3 β; TFEB, Transcription Factor EB; LAMP1, Lysosomal associated membrane protein 1; APAF-1, apoptotic protease activating factor-1; Tfam, Mitochondrial transcription factor A; cTNI, cardiac troponin I; TNF-α, tumor necrosis factor-α; CK-MB, Creatine kinase isoenzyme MB; mPTPs, mitochondrial permeability transition pores; MMP, mitochondrial membrane potential; LDH, lactate dehydrogenase.*

### Coronary Atherosclerotic Heart Disease (CHD)

Coronary atherosclerotic heart disease refers to a heart disease caused by coronary artery atherosclerosis, stenosis, or vascular lumen occlusion that leads to myocardial ischemia, hypoxia, or necrosis (Lyu et al., 2015). Under myocardial ischemia and hypoxia stress caused by atherosclerosis, rapid consumption of creatine kinase, and continuous increase in inorganic phosphate in myocardial cells accelerate glycolysis in cardiomyocytes, increase the production of lactic acid, and promote the production of ATP (Bryk et al., 2017; Kattoor et al., 2017). If the coronary artery is occluded for a long period, intracellular calcium ions are transported into mitochondria through Na^+^/Ca^2+^ exchangers, leading to excessive production of ROS, lengthy abnormal mPTP opening, cell death, protein or cytochrome C release, consumption of a large amount of nucleotides, and degradation of phosphatase and other enzymes and ribozymes, which in turn causes mitochondrial swelling and changes in the membrane potential (Zhang et al., 2017; Orekhov et al., 2019).

Moreover, excessive production of ROS caused by long-term occlusion of the coronary artery can damage mitochondrial DNA, lipids, and proteins and aggravate the production of mitochondrial ROS, leading to a vicious circle that results in vascular endothelial dysfunction and acceleration of atherosclerosis formation (Zinkevich et al., 2017; Zhang M. et al., 2019). In atherosclerosis, the imbalance in mitophagy and mitochondrial fission/fusion leads to the retention of dysfunctional mitochondria, causing mitochondrial energy metabolism disorder and acceleration of myocardial cell apoptosis (Ma et al., 2018; Peng et al., 2020). Therefore, it is imperative to identify safe and effective MQC-regulating natural drugs.

#### Ginsenoside Rg5

Ginsenosides are sterol compounds present in the natural drug ginseng and include Rb1, Rb2, Rg3, and Rg5 (Wang Q. W. et al., 2018; Guo et al., 2020). Ginsenoside Rg5 can improve myocardial ischemia and hypoxia and plays a prominent regulatory role in MQC (Wang et al., 2018; Yuan et al., 2019). Recent studies have shown that Rg5 can enhance the resistance of cardiomyocytes to hypoxia by regulating MQC (Yang et al., 2017a). Its mechanism involves the regulation of mitochondrial hexokinase II (HK-II) and dynamin-related protein 1 (Drp1). Saturated palmitate stimulation can increase lactate accumulation and induce cell acidification by impairing the activity of pyruvate dehydrogenase in cardiomyocytes, leading to the dissociation of HK-II from mitochondria. Rg5 can improve pyruvate dehydrogenase activity, prevent cell acidification, and protect mitochondrial HK-II by inhibiting fatty acid oxidation.

Rg5 can also promote Akt translocation to mitochondria and increase the binding of HK-II to mitochondria, simultaneously inhibiting the collection and fission of Drp1. Using triciribine, an Akt inhibitor, or knocking down Akt expression with small interfering RNA (siRNA) can impair the regulation of mitochondrial function by Rg5, indicating that Rg5 can inhibit Drp1 activation and promote HK-II mitochondrial binding through Akt activation. In addition, Rg5 inhibits mPTPs opening, promotes ATP synthesis, and improves mitochondrial energy metabolism in cardiomyocytes, increasing their resistance to H/R injury. This finding represents a breakthrough in research on Rg5 and its regulation of MQC (Yang et al., 2017a).

#### Ginsenoside Rb1

Ginsenoside Rb1 is another effective active component of ginseng that exerts pharmacological activity towards antioxidant stress and regulates endoplasmic reticulum stress and mitochondrial energy metabolism (Nanao-Hamai et al., 2019; Ye et al., 2019; Zhou et al., 2019). Rb1 can protect cardiomyocytes from hypoxia/ischemia injury *in vitro*, and its protective mechanism primarily involves inhibiting the mitochondrial pathway of apoptosis (Yan et al., 2014). In a previous study, primary neonatal rat cardiomyocytes (NRCMs) were placed in DMEM without glucose and serum and, during hypoxia, Rb1 was administered for 24 h. The damage degree, MMP, and apoptosis degree in NRCMs were determined. The results showed that Rb1 significantly reduced hypoxia/ischemia-induced apoptosis of NRCMs, reduced the transport of cytochrome C from mitochondria to the cytoplasm, restored the level of mitochondrial transmembrane potential, increased the Bcl-2/Bax ratio, and effectively inhibited the activities of caspase-9 and caspase-3 (Yan et al., 2014). These results indicate that Rb1 intervention during *in vitro* hypoxia/ischemia can effectively regulate mitochondrial function and participate in the protection of NRCMs.

#### Berberine (BBR)

Berberine is a quaternary ammonium alkaloid isolated from *Coptis chinensis* as the main active component (Liu et al., 2020). BBR exerts anti-platelet aggregation, stable plaque, and anti-atherosclerotic effects (Li L. et al., 2017; Zhao et al., 2019). *In vitro* studies showed that BBR inhibits adriamycin-induced cardiomyocyte apoptosis by regulating MQC and increasing Bcl-2 expression (Lv et al., 2012). In NRCMs, BBR can inhibit the phosphorylation of adenosine 5’-monophosphate (AMP)-activated protein kinase (AMPK)-α and p53 and expression of cytochrome C and mitochondrial Bax, significantly reduce the loss of MMP induced by doxorubicin (DOX) and inhibit the activity of caspase-3/9. *In vivo* research also showed that BBR not only inhibits caspase-3/9 activation and decreases the phosphorylation level of AMPK-α and p53, but also increases Bcl-2 expression and the survival rate, inhibits cardiac cell apoptosis, and reduces myocardial injury.

Furthermore, BBR can significantly reduce cardiomyocyte damage induced by high glucose, correct the imbalance of mitochondrial fission and fusion, significantly improve mitochondrial function, restore the mitophagy flux of cardiomyocytes by activating the AMPK signaling pathway, and remove damaged mitochondria over time. These results suggest that BBR can promote mitochondrial biosynthesis, restore autophagy flux, and improve high glucose-induced cardiomyocyte injury by activating the AMPK signaling pathway (Hang et al., 2018). Similarly, BBR can improve the level of mitophagy in H9c2 cells under H/R injury, prevent the loss of MMP, reduce mitochondrial dysfunction, and protect cardiomyocytes (Zhu et al., 2020).

#### Orientin (ORI)

Orientin is an effective flavonoid and active component extracted from the natural drug orientin and plays many pharmacological roles as an anti-inflammatory, antithrombotic, antioxidant stress, and myocardial protection compound. Following myocardial ischemia, mPTP opening is the key determinant of cell death. It has been found that the protection exerted by ORI on the myocardium is due to its ability to regulate mitochondrial permeability transition (Lu et al., 2011). Studies have shown that ORI inhibits the abnormal opening of mPTPs, ROS production, excessive cytochrome C release, the levels of Bax and second mitochondria-derived activator of caspase (Smac)/direct IAP-binding protein with low pl (Diablo), and H9c2 cardiomyocyte apoptosis, while increases Bcl-2 levels, and prevented MMP loss. These findings suggest that ORI can regulate mitochondrial function and protect myocardial cells by controlling the closure of the mPTP.

Further, ORI’s ability to regulate mPTPs is inhibited by the phosphatidylinositol 3-kinase (PI3K) inhibitor, wortmannin. This also suggests that the role of ORI in regulating mitochondrial function through mPTPs opening is associated with the PI3K signaling pathway.

### *Periplaneta americana* Extract (PAE)

*Periplaneta americana* is an insect in the genus *Periplaneta*, family Pterygota, and order Periplaneta (Li L. J. et al., 2019). The ethanol extract of the dried insect body exerts antioxidative stress, anti-inflammatory, and anti-tumor pharmacological effects (Chaurasia et al., 2016; Li J. et al., 2019). It has been shown that PTEN-induced kinase 1 (PINK1)/parkin mitotic-mediated PAE can protect H9c2 cardiomyocytes against lipopolysaccharide (LPS)-induced damage (Li J. et al., 2019). The PINK1/parkin pathway is considered as an important route for regulating mitochondrial function, and PINK1 selectively accumulates dysfunctional mitochondria. Parkin and the subsequent parkin-induced recruitment of depolarized mitochondria strictly depend on the mitochondrial targeting signal of PINK1 (Sun et al., 2018).

Experimental results showed that PAE can significantly improve the survival rate of H2c9 cells, inhibit overexpression of the cardiac injury factors cardiac troponin I and creatine kinase isoenzyme (CK-MB) and the inflammation factors interleukin (IL)-1β, IL-6, and tumor necrosis factor (TNF)-α, regulate the expression of mitofusin 1, mitofusin 2, optic atrophy 1 (OPA1), and Drp1, increase the protein and mRNA levels of PINK1 and parkin, inhibit LPS-induced apoptosis, and promote autophagy of myocardial mitochondria. In addition, administration of mitochondrial division inhibitor 1 and Atg7 (autophagy gene) siRNA significantly inhibited the regulatory effect of PAE on mitophagy and myocardial protection (Sun et al., 2018). Overall, these findings show that PAE regulates mitophagy through the PINK1/parkin pathway and protects cardiomyocytes from injury.

### Acute Myocardial Infarction (AMI)

Acute myocardial infarction causes high mortality and represents a critical and severe disease among CVDs (Reed et al., 2017). Several studies have reported that myocardial mitochondrial damage plays an important role in the pathogenesis of AMI (Han et al., 2019; Zhao et al., 2020). Oxidative stress injury caused by mitochondrial energy metabolism disorder and excessive ROS production is one of the main causes of myocardial cell death.

The mitochondria are the primary energy source for myocardial contraction through continuous oxidative phosphorylation (Lu et al., 2010). Under ischemia and hypoxia stress, various signal pathways are activated, which leads to MQC imbalance by affecting uncoupling of the mitochondrial electron transport chain, mPTP opening, and cytochrome C release, further accelerating mitochondrial damage (Jin et al., 2013). The accumulation of ROS and lysosome release caused by mitochondrial damage lead to cardiomyocyte apoptosis and autophagy regulation disorders, which in turn may further affect adjacent myocardial cells and enlarge the infarct area (Disatnik et al., 2013).

Upon acute ischemia, the electrochemical gradient of the mitochondrial intima and respiratory chain activity are destroyed, mitochondrial DNA is damaged, and some functional organelles and proteins are abnormally degraded (Malik and Czajka, 2013; Wang et al., 2017). Damaged mitochondrial DNA is released into the blood after tissue and cell damage, causing an aseptic inflammatory reaction. Therefore, in clinical practice, the level of mitochondrial DNA in the blood circulation of patients with AMI is also significantly increased and positively correlated with the expression of inflammatory factors in the blood (de Haan et al., 2013; Nakahira et al., 2013). Regulation of MQC by natural drugs has thus become an important approach for treating AMI.

#### Que

Que is a flavonoid compound extracted from cherries, onions, and the natural drug bupleurum (Kim D. H. et al., 2019). Its pharmacological effects include the enhancement of capillary resistance, coronary artery dilation, coronary blood flow increase, and anti-tumor activity. Moreover, Que shows a strong antioxidant effect on different cell models (Zhao et al., 2017; Geng et al., 2019; Heger et al., 2019). *In vivo* experimental studies have shown that Que exerts a certain effect on mitochondrial function after myocardial ischemia and reperfusion (Brookes et al., 2002) and can significantly improve mitochondrial energy metabolism and reduce cardiac function damage after I/R in rats.

Furthermore, Que protects H9c2 cardiomyocytes from H_2_O_2_-induced apoptosis (Park et al., 2003), significantly inhibits oxidative stress damage by reducing the production of intracellular ROS, prevents H_2_O_2_-induced mitochondrial antioxidant system dysfunction by regulating mPTP closure, inhibits caspase-3 activation, and regulates the expression of Bcl-2 (Park et al., 2003). These results suggest that Que protects H9c2 cardiomyocytes from oxidative damage by regulating MQC and inhibiting caspase activity.

In addition, vascular calcification is a strong independent predictor of the incidence rate of CVDs and increased mortality (Phadwal et al., 2020). Que prevents mitochondrial lysis by inhibiting oxidative stress and regulating Drp1 phosphorylation, increases ATP synthesis and MMP, and alleviates apoptosis and calcification of vascular smooth muscle cells induced by inorganic phosphate. An *in vivo* study also reported that Que improves adenine-induced aortic calcification (Cui et al., 2017). These results indicate that Que can reduce vascular smooth muscle cell apoptosis by reducing oxidative stress, inhibiting mitochondrial cleavage, and reducing calcification and the incidence rate of CVDs.

#### Tanshinone II-A

Tanshinone II-A is the most effective active component in *Salvia miltiorrhiza* and improves microcirculation, dilates coronary arteries, and reduces platelet adhesion and aggregation (Li L. et al., 2017; Liang et al., 2018; Cheng et al., 2019). Tanshinone II-A can be used to treat hypoxia-induced mitochondrial dysfunction in H9c2 cells by regulating mitochondrial ROS, intracellular nitric oxide (NO), and calcium levels (Jin and Li, 2013). It has been reported that hypoxia significantly reduces the viability of cardiomyocytes, promotes LDH and ROS production, increases the levels of NO and Ca^+^, and inhibits SOD activity and mitochondrial ATP synthesis. Tanshinone II-A significantly reversed the abovementioned effects of hypoxia, suggesting that its protective effect on H9c2 cardiomyocytes is related to regulation of the mitochondrial antioxidant system.

Tanshinone II-A can also reduce myocardial I/R injury by regulating SIRT1-peroxisome proliferator-activated receptor gamma coactivator 1-α (PGC1α) (Zhong et al., 2019). *In vivo* studies have suggested that I/R can mediate microvascular wall damage, lumen stenosis, perfusion defects, and cardiac microvascular endothelial cell (CMEC) apoptosis by inducing mitochondrial damage. Tanshinone II-A can maintain the activity and microvascular homeostasis of CMECs and reduce myocardial microvascular injury (Li S. et al., 2017). Additionally, *in vitro* studies indicated that tanshinone II-A can activate the SIRT1-PGC1α signaling pathway, maintain the MMP level, reduce the expression of mitochondrial pro-apoptotic factors, inhibit abnormal mPTP opening, block mitochondrial apoptosis, and provide a good living environment for CMECs. In contrast, inhibiting the SIRT1-PGC1α signaling pathway reduces the beneficial effects of tanshinone II-A on mitochondrial function regulation, CMEC survival, and myocardial microvascular homeostasis. These results show that tanshinone II-A protects myocardial microvessels by activating the SIRT1-PGC1α pathway. *In vivo* studies also found that tanshinone II-A inhibits abnormal mPTP opening in a dose-dependent manner and reduces the size of myocardial infarction (Zhang et al., 2005).

#### Gastrodin (Gas)

Gastrodin is the most abundant active component extracted from the dried rhizome of *Gastrodia elata* and can produce anti-inflammatory and antioxidative stress pharmacological effects to increase the myocardial blood supply and regulate vasomotor function (Li and Zhang, 2015; Liang W. Z. et al., 2017; Liu et al., 2018; Yan J. et al., 2019). Recent studies have shown that Gas protects H9c2 cardiomyocytes against oxidative stress injury, primarily through the regulation of mPTPs (Han et al., 2018). Gas can also inhibit MMP decrease induced by oxidative stress, prevent the loss of membrane potential, significantly reduce ROS excessive production and caspase-3 overexpression induced by H_2_O_2_, and reduce cardiomyocyte apoptosis. This suggests that Gas reduces oxidative stress-induced H9c2 cell injury by inhibiting mPTPs opening.

Furthermore, Gas can protect H9c2 cardiomyocytes from oxidative stress by improving mitochondrial dynamics and mitochondrial dysfunction (Cheng et al., 2020). H_2_O_2_ induces mitochondrial ROS production, inhibits the respiratory function of mitochondria and mitofusin-2 (Mfn2) and OPA1 expression, and increases mitochondrial fission 1 protein expression. In contrast, Gas can promote the nuclear transport of Nrf2 induced by H_2_O_2_, increase Mfn2 and OPA1 expression, inhibit ROS production and mitochondrial fission 1 protein overexpression, improve mitochondrial respiratory function and ATP production, and protect H9c2 cardiomyocytes from oxidative stress. Thus, the mechanism of Gas underlying the protection of H9c2 cardiomyocytes may also rely on increased nuclear displacement of Nrf2, regulation of mitochondrial dynamics, and the maintenance of mitochondria quality and function.

#### Astragaloside IV (AST)

Astragaloside is extracted from the dried root of *Astragalus membranaceus*. As a drug commonly used to treat CVDs, AST exerts anti-inflammatory and antioxidant effects, enhances immunity, lowers blood pressure, and regulates MQC (Jiang et al., 2019; Lin et al., 2019; Wang et al., 2019). In addition, AST can reduce I/R-induced apoptosis by inhibiting activation of the death receptor pathway and key mitochondrial pathway factors (Yin et al., 2020) and induce isolated heart and myocardial cells to resist ischemia-induced stress injury by improving Bcl-2-mediated mitochondrial function (Luo et al., 2019). AST significantly upregulates the expression of Bcl-2, particularly in the mitochondria of cardiomyocytes, and inhibits mitochondrial ROS generation, maintains MMP, regulates mPTPs opening, inhibits H9c2 cardiomyocyte apoptosis, promotes the recovery of rat myocardial function, and reduces the area of myocardial infarction. These results suggest that AST may modulate MQC by upregulating Bcl-2 and promoting its translocation to mitochondria, maintaining MMP, and inhibiting the cascade events induced by ROS, thus preventing mPTP opening, inhibiting cardiomyocyte opening, and alleviating myocardial injury. Notably, AST was found to protect H9c2 cardiomyocytes from oxidative stress by inactivating glucogen synthase kinase-3 beta through NO (He et al., 2012).

Furthermore, AST has been reported to inhibit DOX-induced cardiomyocyte apoptosis mediated by the mitochondrial pathway of apoptosis by regulating the PI3K/Akt pathway to restore the beating rate of cardiomyocytes and significantly improve DOX-induced cardiomyocyte dysfunction. In addition, a study has demonstrated that AST significantly reduces ROS production and LDH, CK-MB, and cytochrome C release induced by DOX and restores mitochondrial function (Jia et al., 2014). The previous data (Jia et al., 2014)suggest that AST protects cardiomyocytes by regulating the mitochondrial antioxidant system and, consequently, MQC.

#### Tetramethylpyrazine (TMP)

Tetramethylpyrazine is an amide alkaloid isolated and purified from *Ligusticum wallichii* and displays antioxidative stress, anti-platelet aggregation, and anti-thrombosis effects (Wang G. et al., 2016; Li J. et al., 2019; Yan J. et al., 2019). Recent studies have also found that TMP can effectively improve LPS-induced cardiomyocyte injury in neonatal rats, with the main underlying regulatory mechanism being the regulation of MQC (Huang B. et al., 2018). The protective effect of TMP on LPS-induced myocardial injury may be achieved by upregulation of 14-3-3 γ, a well-known protector of LPS-induced myocardial injury, and control of mitochondrial quality. In fact, TMP can upregulate the expression of mitochondrial 14-3-3 γ and Bcl-2, activate the phosphorylation of Bad (S112), increase cell viability and MMP, reduce LDH and caspase-3 activity, inhibit ROS production, abnormal mPTPs opening, and cardiomyocyte apoptosis rate, and reduce primary myocardial cell damage induced by LPS. The cardioprotective effect of TMP is attenuated by pad/14-3-3 γ-short hairpin RNA, an adenovirus that inhibits the expression of 14-3-3 γ (Huang B. et al., 2018). Therefore, the protective effect of TMP on LPS-induced myocardial injury is achieved by upregulating 14-3-3 γ expression, promoting the translocation of Bcl-2 to mitochondria, regulating abnormal mPTPs opening, and improving mitochondrial function in myocardial cells.

### Heart Failure (HF)

Heart failure is a clinical syndrome caused by ventricular filling or ejection dysfunction induced by abnormal cardiac structure and function (Braunwald, 2013; Metra and Teerlink, 2017). Abnormal mitochondrial energy metabolism is an important factor in the development of HF, directly or indirectly affecting the physiological function of cardiomyocytes by regulating bioenergy, redox, oxidative stress, excitation-contraction coupling, and apoptosis (Lee and Tian, 2015). The mitochondrial respiratory chain can provide energy for the heart muscle (Tian R. et al., 2019); however, when its function is abnormal or energy metabolism disorder occurs, an excessive amount of ROS is produced, which causes damage to the mitochondrial structure and function, further deteriorating myocardial energy utilization and myocardial function (Huss and Kelly, 2005; Song et al., 2014). In HF, an abnormal working efficiency of the mitochondrial respiratory chain in cardiomyocytes leads to electron leakage during transport. Moreover, ROS production in mitochondria increases, triggering mitochondrial channels and endomembrane ion channels. Abnormal opening of mPTPs results in abnormal MMP, further aggravating the production of mitochondrial ROS and oxidative stress damage (Dolinsky et al., 2016; Sun and Yang, 2017a).

Additionally, the decrease in autophagy and increase in response sensitivity damage mitochondrial respiratory chain function (Dolinsky et al., 2016). Mitophagy dysfunction in HF may lead to further deterioration of myocardial energy metabolism. Therefore, targeting MQC in myocardial cells is an effective treatment strategy against HF (Chu et al., 2013).

#### Resveratrol (Res)

Resveratrol is a natural antioxidant with strong biological activity, widely found in *Polygonum cuspidatum*, mulberry, grape, peanut, and other plants (Li T. et al., 2019). Its activity includes anti-inflammatory and antioxidative stress abilities, inhibition of platelet aggregation and thrombosis, and regulation of MQC (Haramizu et al., 2017; Folbergrova et al., 2018; Sedlak et al., 2018). Res can significantly regulate the expression of Drp1, improve mitochondrial elongation, and increase the translocation of parkin and PINK1. Simultaneously, LC3-II expression is significantly increased by Res, and damaged mitochondria of aging cardiomyocytes are degraded. These findings suggest that inhibition of mitochondrial elongation in a Drp1-dependent manner is related to the effect of Res on the development of senescent cardiomyocytes and that activation of parkin and PINK1 are the basis of the mechanism by which Res regulates MQC and protects aging cardiomyocytes (Ren et al., 2017).

In addition, Res exerts protective effects against H/R-induced oxidative stress and the mitochondrial pathway of apoptosis on NRCMs. Res can significantly reduce the disturbance of α-actin and F-actin caused by H/R injury, improve the structural damage of NRCMs, and regulate the ratio of Bcl-2/Bax and activity of caspase-3. Moreover, it can inhibit mitochondrial oxidative stress and cardiomyocyte apoptosis induced by H/R injury (Li J. et al., 2019).

Res also regulates MQC by activating the SIRT1 signaling pathway to reduce cardiac dysfunction in diabetic cardiomyopathy mice (Ma et al., 2017). SIRT1 regulates mitochondrial dynamics and protects from dilated cardiomyopathy (DCM). Accordingly, SIRT1 gene knockout mice show DCM symptoms post modeling. Res can inhibit cardiomyocyte apoptosis by activating SIRT1 to reverse DCM in mice. In addition, SIRT1 function is mediated by the deacetylation of PGC-1α. Therefore, Res likely activates SIRT1 through PGC-1α-mediated mitochondrial regulation to improve DCM myocardial injury.

### *Panax notoginseng* Saponins (PNS)

*Panax notoginseng* saponins are extracted from *P. notoginseng* (Liang X. et al., 2017). It exhibits antioxidative stress, anti-myocardial apoptosis, anti-inflammatory, and hypolipidemic properties, and can improve microcirculation and regulate endothelial cell function (Hu et al., 2018; Zhang M. et al., 2019; Wang et al., 2020a). PNS were found to inhibit cardiomyocyte apoptosis of naturally aging rats through the mitochondrial pathway (Zhou H. et al., 2018). PNS can significantly improve the morphological and pathological changes in the myocardium of aging rats, reverse the downregulation of Forkhead box O3 and manganese superoxide dismutase, inhibit the activity of malondialdehyde (MDA), upregulate PGC-1α, LC3-β, and beclin-1, and restore mitophagy flow. PNS can also inhibit increases in cardiomyocyte apoptosis and improve mitochondrial dysfunction caused by aging in a dose-dependent manner (Zhou H. et al., 2018). This study also reported that during the process of natural aging, mitochondrial dysfunction leads to further increases in oxidative damage, which plays a key regulatory role in cardiomyocyte apoptosis. PNS can attenuate oxidative damage through oxidative stress- and mitochondrial function-related signaling pathways, thus exerting an anti-apoptotic effect. This highlights that MQC is essential in myocardial apoptosis protection.

#### Luteolin (LTL)

Luteolin is a plant-derived flavonoid that exists in *Buddleja officinalis*. Its several pharmacological activities include anti-inflammatory, antioxidative stress, and anti-angiogenesis abilities and regulation of MQC (An et al., 2016; Ou et al., 2019; Chen H. I. et al., 2020). LTL was found to improve adriamycin-induced cardiac toxicity and cardiac contractile dysfunction, and its therapeutic effect is primarily associated with the regulation of mitophagy. This compound can significantly improve doxorubicin-induced myocardial contractile dysfunction, including the increase in the peak shortening amplitude and maximum shortening/lengthening rate. LTL also inhibits excessive ROS production induced by doxorubicin and prevents the loss of MMP. In addition, LTL was shown to increase the level of mitophagy, promote Drp1 phosphorylation and transcription factor EB expression, and weaken mitochondrial elongation induced by low-dose doxorubicin. Following the administration of mitochondrial division inhibitor 1, a Drp1 GTPase inhibitor, LTL inhibits the regulation of transcription factor EB, lysosomal associated membrane protein 1, and LC3-II, resulting in serious MMP loss and myocardial contractile dysfunction (Xu et al., 2020). These findings provide insight into the protective mechanism of LTL in cardiomyocytes.

#### Catalpol (CTL)

Catalpol is an iridoid glucoside derived from *Rehmannia glutinosa* that exerts anti-cancer, neuroprotective, anti-inflammatory, antioxidant stress, and mitochondrial function regulatory effects (Jiang and Zhang, 2020; Yan et al., 2020; Zhang et al., 2020). CTL can inhibit H_2_O_2_-induced cardiomyocyte apoptosis through the mitochondria-dependent caspase pathway. In addition, CTL protects H9c2 cells from H_2_O_2_-induced cytotoxicity and apoptosis, significantly reduces MDA release, and increases SOD activity. These findings show that CTL pretreatment protects H9c2 cells against H_2_O_2_-induced apoptosis, and its protective effect is associated with the mitochondria-dependent caspase pathway, which is in turn associated with increased Bcl-2 and decreased Bax expression (Hu et al., 2016).

### Myocardial I/R Injury

Myocardial I/R injury refers to the process of regaining blood perfusion during a certain period of time post partial or complete acute coronary artery obstruction (Ibanez et al., 2015; Botker, 2019). Due to the myocardial ultrastructure damage caused by acute ischemia, the energy metabolism of mitochondria in myocardial cells becomes abnormal and the physiological function of ion channels is disturbed (Liu Y. F. et al., 2017), which is more prominent after I/R injury, leading to large-area myocardial infarction and even sudden death (Cadenas, 2018; Boengler et al., 2019).

Mitochondrial quality control plays an important role in the pathogenesis of myocardial I/R injury (Cai et al., 2018; Wang et al., 2020b). Hypoxia, ischemic stress response, and secondary injury of reperfusion can directly affect the closing function of mPTPs, which may be an important mechanism leading to myocardial I/R injury. During myocardial ischemia, the accumulation of calcium, long-chain fatty acids, and ROS can open mPTPs (Cai et al., 2020). During the period in which myocardial hypoxia occurs, anaerobic fermentation can increase the production of lactic acid, reduce the pH of local blood, and inhibit the opening of mPTPs. Moreover, during the reperfusion phase, the respiratory function of mitochondria is restored, and the transmembrane potential undergoes repair. When the respiratory chain regains oxygen, it then produces a large amount of ROS, and calcium overload promotes abnormal mPTPs opening (Morciano et al., 2017). The latter destroys the electrochemical proton gradient on both sides of the mitochondrial inner membrane, the coupling of oxidized phosphoric acid is dissolved, and ATP synthesis becomes dysfunctional (Chen Q. et al., 2020). Moreover, some macromolecular proteins in the mitochondrial matrix cannot pass through mPTPs, and thus the osmotic pressure in the matrix becomes relatively high. All types of ions, water, and other molecules enter the mitochondrial matrix non-selectively, which causes mitochondrial swelling and outer membrane rupture (Li L. et al., 2019). Simultaneously, apoptotic factors, such as cytochrome C, and apoptosis-inducing factors enter the cytoplasm through the membrane space, causing cardiomyocyte apoptosis (Wang G. et al., 2016; Xu et al., 2019).

Therefore, MQC is particularly important for treating myocardial I/R injury. Indeed, that many natural drugs and effective active ingredients can regulate MQC in the I/R stage, alleviating mitochondrial damage and myocardial cell apoptosis caused by I/R.

#### Diosgenin (DSG)

Diosgenin is an important active component of steroidal saponins from *Dioscorea opposita* Thunb. Recent studies have shown that DSG displays efficacious antioxidant stress, anti-inflammatory, and hypolipidemic pharmacological effects (Mischitelli et al., 2016; Kiasalari et al., 2017; Sethi et al., 2018). *In vivo*, DSG blocks the mitochondrial ATP-sensitive potassium channel (mitokatap) and NO system (Badalzadeh et al., 2015). Following DSG treatment, left ventricular diastolic blood pressure and systolic force were significantly improved and restored to the levels achieved before ischemia. Blocking mitokatap with 5-hydroxydecanoate completely blocked the regulatory effect of DSG on mitochondrial function and improvement of left ventricular diastolic pressure and systolic force. In addition, after blocking of the NO system with nitroso-l-arginine methyl ester, the therapeutic effect of DSG decreases, and its inhibitory effect becomes less potent than that under 5-hydroxydecanoate treatment(Badalzadeh et al., 2015). These results suggest that DSG exerts a protective effect against myocardial reperfusion injury by regulating mitokatap and the NO system.

Additionally, DSG significantly reduces LDH release into the coronary effluent and significantly inhibits IL-6, IL-1β, and TNF-α expression during reperfusion, as well as improves myocardial contractility. 5-Hydroxydecanoate inhibited mitokatap and significantly reversed the myocardial protective effect of DSG (*P* < 0.05), confirming that DSG reduces the production of inflammatory mediators and improves myocardial contractility by activating mitokatap (Ebrahimi et al., 2014).

Moreover, DSG can play an anti-arrhythmic role by regulating mitokatap (Badalzadeh et al., 2014). Administration of DSG before ischemia reduces LDH release into the coronary outflow. Following reperfusion, DSG can reduce the number of ventricular tachycardia, ventricular fibrillation, and premature ventricular contractions in rats with myocardial I/R injury, shorten the duration of ventricular tachycardia and ventricular fibrillation, and significantly improve arrhythmia during reperfusion.

#### Lycopene (LYP)

Lycopene is a carotenoid acting as a natural antioxidant in tomato. Due to its strong antioxidant effect, LYP shows potential for use in the treatment of CVDs and cerebrovascular diseases associated with oxidative damage (Qu et al., 2016; Fan et al., 2019; Sun et al., 2019). A study has shown that LYP exerts a strong protective effect against myocardial I/R injury, which is primarily achieved by mPTPs regulation (Li J. et al., 2019). LYP can effectively inhibit abnormal mPTPs opening and cytochrome C, apoptotic protease activating factor-1, and caspase-3/9 overexpression (Li X. et al., 2019). Moreover, this carotenoid can significantly increase the expression of Bcl-2 and decrease that of Bax. Additionally, LYP can increase the survival rate of myocardial cells, reduce their apoptosis rate, and reduce the area of myocardial infarction. However, the protective effect of LYP against myocardial I/R injury was eliminated by atractyloside (Regulatory drugs promoting the opening of mPTPs). Therefore, LYP may improve myocardial I/R injury by inhibiting mPTPs opening and regulating the Bcl-2/Bax ratio.

Further *in vivo* and *in vitro* experiments have shown that I/R injury increases the content of mt 8-hydroxyguanine, decreases the mitochondrial DNA content and DNA transcription level, and induces mitochondrial dysfunction and cardiomyocyte apoptosis (Yue et al., 2015). LYP was reported to inhibit the production of ROS in mitochondria, decrease the activity of MDA, increase mitochondrial transcription factor A protein levels, restore the level of MMP and ATP synthesis, and inhibit myocardial apoptosis and the myocardial necrosis area (Yue et al., 2015). Thus, the protective effect of LYP on mitochondrial DNA is related to decreased ROS production and stabilization of mitochondrial transcription factor A.

Therefore, LYP can protect cardiomyocytes and mitochondrial DNA from oxidative stress-induced by I/R injury. In addition, LYP improves H/R-induced NRCM apoptosis, as LYP pretreatment inhibits the activation of mPTPs by reducing the ROS level in cardiomyocytes and inhibiting the increase in MDA levels, which protects rats against myocardial cell damage under hypoxia stress (Yue et al., 2012).

### Curcumin (Cur)

Curcumin is a polyphenol extracted from *Curcuma longa* that acts as an anti-oxidant and anti-inflammatory agent and regulates immunity and mitochondrial energy metabolism (Daverey and Agrawal, 2016; Farzaei et al., 2018; He et al., 2018). Cur was found to reduce mitochondrial oxidative stress injury induced by myocardial I/R injury in rats by activating SIRT1 (Yang et al., 2013). In the absence of sirtinol (a SIRT1 inhibitor) or SIRT1 siRNA, Cur displays strong MQC regulation ability and myocardial protection. Cur can maintain the mitochondrial redox potential, increase SOD activity, and reduce the production of H_2_O_2_ and malondialdehyde. It can also significantly upregulate Bcl-2, downregulate Bax, reduce myocardial apoptosis and the myocardial infarction area, and improve cardiac function after myocardial ischemia. The effectiveness of Cur on regulating mitochondrial function can be reversed by sirtinol or SIRT1 siRNA treatment, indicating that Cur improves mitochondrial oxidative damage induced by I/R through SIRT1 signaling, thus protecting myocardial cells and myocardial tissue.

Furthermore, Cur can improve bevacizumab (BEV)-induced myocardial mitochondrial dysfunction by inhibiting oxidative stress (Sabet et al., 2020). BEV induces mitochondrial ROS overproduction, MMP collapse, mitochondrial swelling and deformation, and cardiomyocyte apoptosis. Cur can significantly improve mitochondrial toxicity induced by BEV, inhibit ROS production, reduce MDA activity, restore MMP levels, and improve myocardial mitochondria function. These findings suggest that a combination of Cur and BEV can protect myocardial mitochondria from BEV-induced damage and provide a reliable basis for the mechanism of natural drugs underlying the reduction of drug-triggered toxicity.

#### Icariin (ICA)

Icariin is an active component of dried stems and leaves of *Epimedium* species (Huang Z. et al., 2018). It increases blood vessel flow, promotes hematopoietic function, increases immune regulation, and exhibit antioxidant properties (Fang and Zhang, 2017; Huang B. et al., 2018; Mi et al., 2018). ICA can inhibit I/R-induced mitochondrial oxidative damage, reduce the MDA content, increase SOD activity, significantly improve myocardial contractile function after I/R, reduce myocardial CK-MB and LDH leakage, and reduce the myocardial infarction area. This compound can also improve the stability of mitochondria by increasing MMP, thus further inhibiting cardiomyocyte apoptosis (Wu et al., 2018). In addition, sirtuin-1 is upregulated and FOXO1 is downregulated upon ICA administration. Sirtinol and SIRT1 siRNA can block the regulation ability of ICA and destroy ICA-mediated mitochondrial homeostasis. This suggests that ICA regulates mitochondrial function under oxidative stress by activating sirtuin-1/FOXO1 signaling, which protects cardiomyocytes from I/R-induced oxidative stress.

Furthermore, ICA protects myocardial cells against apoptosis associated with hypertension through the mitochondrial pathway of apoptosis and can increase the expression of Bcl-2, reduce that of p53, Bax, and caspase-3, inhibit cardiomyocyte apoptosis, improve mitochondrial dysfunction and left ventricular remodeling of myocardial cells, and reduce the blood pressure in model rats (Qian et al., 2017).

## Discussion

**FIGURE 2:**
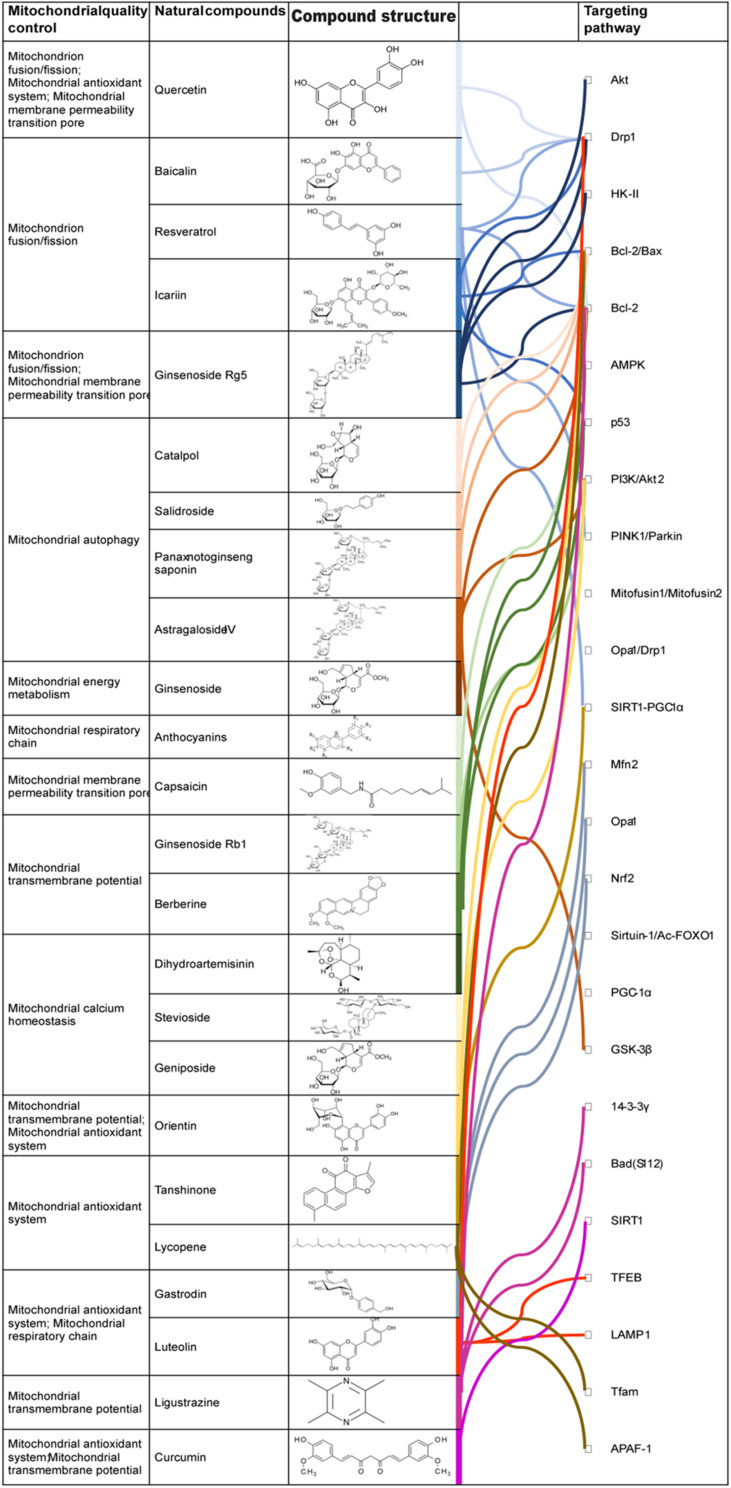
Regulationmechanism of Natural drugs in MQC. Active ingredients in natural drugs can influence the production of energy-supplying substances in the mitochondria, interfere with the expression of genes associated with mitochondrial energy requirements, and regulate various mechanisms of MQC modulation.

Mitochondria are the primary source of energy of myocardial cells. The quality and quantity of mitochondria must be strictly controlled to ensure their normal function, as well as that of cardiomyocytes. Upon mitochondrion fission/fusion, autophagy, and mitochondrial energy metabolism dysfunction, cardiomyocytes suffer oxidative stress, apoptosis, and abnormal autophagy. As shown as Figure 2, in CVDs, particularly atherosclerosis, ischemic cardiomyopathy, hypertrophic cardiomyopathy, HF, and AMI, mitochondrion fission/fusion imbalance and mitophagy disorders are important mechanisms of MQC impairment. Nonetheless, the initiation mechanism of mitochondrial dysfunction in CVDs has not been fully elucidated. With further research on this topic, the important contribution of MQC to CVDs will be gradually revealed, providing the opportunity for new approaches to experimental research and clinical treatment of CVDs.

In-depth and systematic studies of the mechanism of MQC in CVDs as well as large-scale, multi-center, large-scale clinical research and evidence-based medicine research have not been performed. However, *in vivo* and *in vitro* experimental results on different diseases have clearly demonstrated that various natural drugs can have various MQC-related pharmacological activities by directly or indirectly regulating mitochondrial function. These experimental results not only improve the understanding of the effective mechanisms of the active ingredients in natural drugs, but also identify scientific challenges that should be addressed in the future. Currently, there are three scientific challenges to require urgent attention.

(1) Natural drugs do not exert their full efficacy easily. Natural drugs and active ingredients can directly or indirectly regulate MQC, exerting their pharmacological effects. However, the passive diffusion of some small molecules hinders their penetration of the mitochondrial double-membrane, which functions as a key barrier for natural drugs and prevents these drugs from exerting their roles. The pharmacodynamic effects of some natural drugs are mostly limited to the regulation of mitochondrial function through multiple signaling pathways or regulation of the antioxidant defense and mPTPs opening and closing. Therefore, to ensure that natural drugs function as expected in MQC, it is necessary to identify more mitochondria-targeted natural drugs or to assist them in efficiently exerting their effects in mitochondria function through nanotechnology.

(2) Few studies have examined mitochondrial dynamics. Research on the myocardial cell mechanics in CVDs has demonstrated that their automaticity/rhythmicity, excitability, conductivity, and contractility play a key role in their mechanics and diseases. The distribution and transport of mitochondria is at the core of cardiac function. Research on the role of natural drugs in MQC has illustrated their role regulating mitochondrial ROS production, mitochondrial respiratory function, and mPTPs opening and closing. However, few studies have examined the effects of natural drugs on mitochondrial dynamics, interaction between mitochondria and other organelles, and relationship between mitochondria and endoplasmic reticulum stress, as well as other biological processes.

(3) The specific targets of most natural drugs that regulate MQC remain unclear. Natural drugs control mitochondrial quality primarily by regulating mPTPs’ activity, respiratory chain enzymes, key enzymes of the tricarboxylic acid cycle, and antioxidant kinases. Most studies have focused on the effect of natural drugs on mitochondrial function, whereas only few studies have evaluated the targets of these drugs for MQC regulation and their mechanism of antagonistic action. Therefore, mitochondrial proteomics, chromatography-mass spectrometry, and other techniques should be used to screen possible target sites and determine the cause of underlying the antagonizing action mechanism of natural drugs.

## Conclusion

In conclusion, this review discussed the regulatory roles of natural drugs in mitophagy, mitochondrial fission and fusion, mitochondrial energy metabolism, the mitochondrial respiratory chain, and mPTPs from the perspective of MQC. We have also summarized the regulatory mechanisms of a variety of natural drugs on MQC and their protective role toward cardiomyocytes in CHD, AMI, myocardial I/R injury, and HF. Research on natural drugs regulating MQC in CVDs is currently being conducted on a large scale. Although several urgent challenges remain to be addressed, the material basis of natural drugs influencing mitochondrial function and specific mechanisms and targets of natural drugs in MQC regulation will be clarified in the future. This evidence will facilitate the discovery of new drugs or lead compounds for treating CVDs.

The mitochondria play an important role in energy production, signal transduction, Ca2+ homeostasis, and apoptosis regulation in cardiomyocytes thereby significantly influencing cardiac function and blood circulation. The mechanisms underlaying mitochondrial dysfunction are complex, diverse, and interconnected. Mitochondrial dysfunction influences the occurrence and development of various cardiovascular diseases, but its onset remains unclear; the interaction between oxidative stress and autophagy may possibly be one underlaying mechanism of such onset. Natural antioxidants, which can regulate mitochondrial dysfunction, may potentially be a novel therapeutic strategy against CVD in the future.

## Author Contributions

XC, TZ, and PY defined the research theme. WZ, CM, and ZZ searched for the related articles. QM, YZ, LZ, TZ, and XC collated all related articles. XC wrote the manuscript. All authors commented on the manuscript.

## Conflict of Interest

The authors declare that the research was conducted in the absence of any commercial or financial relationships that could be construed as a potential conflict of interest.
