# Supplementary figures and images for: Regulation of Mitochondrial Quality Control by Natural Drugs in the Treatment of Cardiovascular Diseases: Potential and Advantages
Source: Front Cell Dev Biol. 2020 Dec 23;8:616139. doi: 10.3389/fcell.2020.616139 (PMC7793684; doi:10.3389/fcell.2020.616139)

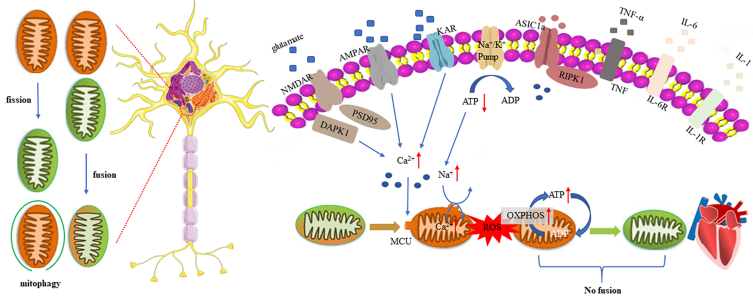

Supplement: Supplementary file 1 [file Data_Sheet_1.PDF]
