# Supplementary material for: Regulation of Mitochondrial Quality Control by Natural Drugs in the Treatment of Cardiovascular Diseases: Potential and Advantages
Source: Front Cell Dev Biol. 2020 Dec 23;8:616139. doi: 10.3389/fcell.2020.616139 (PMC7793684; doi:10.3389/fcell.2020.616139)

Mitochondrialquality control	Naturalcompounds	Compound structure
Mitochondrion fusion/fission; Mitochondrial antioxidant system; Mitochondrial membrane permeability transition pore	Quercetin	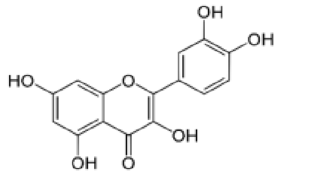
Mitochondrion fusion/fission	Baicalin	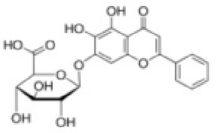
	Resveratrol	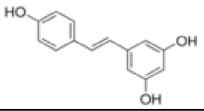
	Icariin	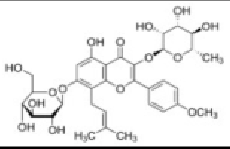
Mitochondrion fusion/fission; Mitochondrial membrane permeability transition pore	Ginsenoside Rg5	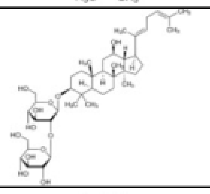
Mitochondrial autophagy	Catalpol	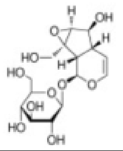
	Salidroside	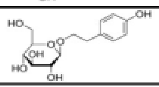
	Panaxnotoginseng saponin	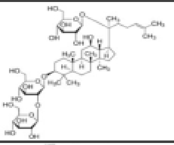
	AstragalosideIV	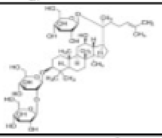
Mitochondrial energy metabolism	Ginsenoside	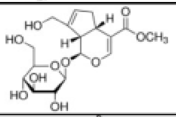
Mitochondrial respiratory chain	Anthocyanins	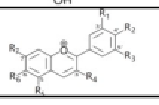
Mitochondrial membrane permeability transition pore	Capsaicin	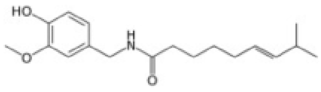
Mitochondrial transmembrane potential	Ginsenoside Rb1	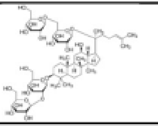
	Berberine	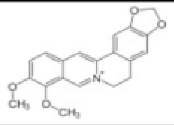
Mitochondrial calcium homeostasis	Dihydroartemisinin	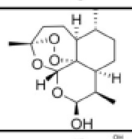
	Stevioside	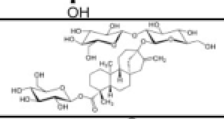
	Geniposide	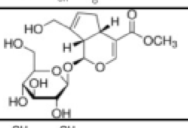
Mitochondrial transmembrane potential; Mitochondrial antioxidant system	Orientin	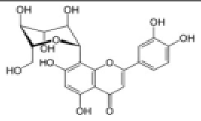
Mitochondrial antioxidant system	Tanshinone	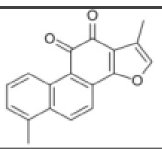
	Lycopene	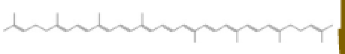
Mitochondrial antioxidant system; Mitochondrial respiratory chain	Gastrodin	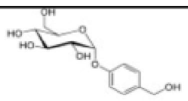
	Luteolin	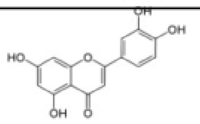
Mitochondrial transmembrane potential	Ligustrazine	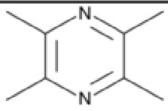
Mitochondrial antioxidant system;Mitochondrial transmembrane potential	Curcumin	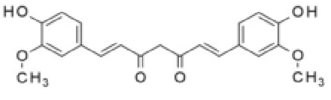

Targeting pathway

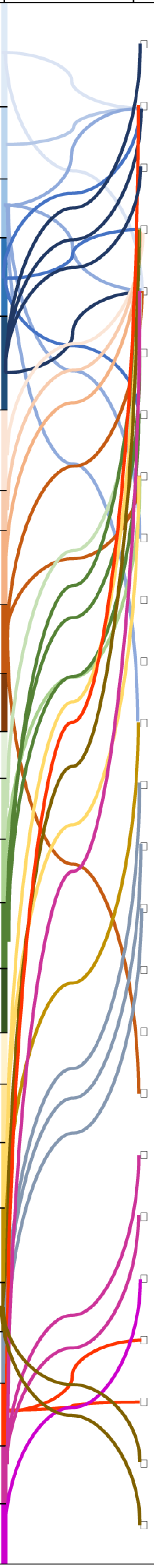

Supplement: Supplementary file 2 [file Data_Sheet_2.PDF]
